# Cell death pathways: molecular mechanisms and therapeutic targets for cancer

**DOI:** 10.1002/mco2.693

**Published:** 2024-09-04

**Authors:** Shaohui Wang, Sa Guo, Jing Guo, Qinyun Du, Cen Wu, Yeke Wu, Yi Zhang

**Affiliations:** ^1^ State Key Laboratory of Southwestern Chinese Medicine Resources, School of Ethnic Medicine Chengdu University of Traditional Chinese Medicine Chengdu China; ^2^ State Key Laboratory of Southwestern Chinese Medicine Resources, School of Pharmacy Chengdu University of Traditional Chinese Medicine Chengdu China; ^3^ College of Clinical Medicine Hospital of Chengdu University of Traditional Chinese Medicine Chengdu China

**Keywords:** apoptosis, autophagy, cancer therapy, cell death, drug resistance, ferroptosis, necroptosis, pyroptosis, tumor microenvironment

## Abstract

Cell death regulation is essential for tissue homeostasis and its dysregulation often underlies cancer development. Understanding the different pathways of cell death can provide novel therapeutic strategies for battling cancer. This review explores several key cell death mechanisms of apoptosis, necroptosis, autophagic cell death, ferroptosis, and pyroptosis. The research gap addressed involves a thorough analysis of how these cell death pathways can be precisely targeted for cancer therapy, considering tumor heterogeneity and adaptation. It delves into genetic and epigenetic factors and signaling cascades like the phosphatidylinositol 3‐kinase/protein kinase B/mammalian target of rapamycin (PI3K/AKT/mTOR) and mitogen‐activated protein kinase/extracellular signal‐regulated kinase (MAPK/ERK) pathways, which are critical for the regulation of cell death. Additionally, the interaction of the microenvironment with tumor cells, and particularly the influence of hypoxia, nutrient deprivation, and immune cellular interactions, are explored. Emphasizing therapeutic strategies, this review highlights emerging modulators and inducers such as B cell lymphoma 2 (BCL2) homology domain 3 (BH3) mimetics, tumour necrosis factor‐related apoptosis‐inducing ligand (TRAIL), chloroquine, and innovative approaches to induce ferroptosis and pyroptosis. This review provides insights into cancer therapy's future direction, focusing on multifaceted approaches to influence cell death pathways and circumvent drug resistance. This examination of evolving strategies underlines the considerable clinical potential and the continuous necessity for in‐depth exploration within this scientific domain.

## Graphical abstract

This graphical abstract provides a comprehensive view of therapeutic strategies for studying cell death pathways in cancer.

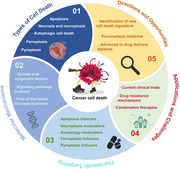

## 1 INTRODUCTION

Cancer remains a formidable global health challenge, largely due to its complex biology and the ability to adapt and resist traditional treatment methods. Historically, understanding of cancer has expanded from not only seeing it as a disease characterized by uncontrolled cell growth but also involving aberrant cell death mechanisms that are crucial for tumor sustenance and progression.^1^, ^2^ The dysregulation of cell death pathways is central in cancer pathogenesis, promoting tumor development while offering potential therapeutic targets.^3^, ^4^ Importantly, the subversion of programmed cell death by cancer cells plays a critical role in tumor initiation, progression, and their resistance to therapies, making it imperative to understand and control these pathways.^5^

Cellular homeostasis necessitates a balance between cell proliferation and cell death.^6^ In malignancies, this balance is disrupted, skewing toward proliferation largely due to impaired cell death mechanisms.^7^, ^8^ Apoptosis, once considered the sole programmatic avenue of cell death, is critically involved in preventing cancer by eliminating defective cells potentially harboring oncogenic mutations.^7^ However, cancer cells frequently acquire mutations that enable them to circumvent apoptotic signaling, contributing to tumor survival and growth.^9^ Besides apoptosis, emerging aspects of other cell death forms like necroptosis, autophagy, ferroptosis, and pyroptosis each contribute uniquely to cancer dynamics, influencing not only tumor cell fate but also the tumor microenvironment (TME) and immune surveillance.^10^, ^11^, ^12^, ^13^, ^14^, ^15^ These alternative forms of cell death offer a breadth of therapeutic possibilities.^16^, ^17^ For instance, the selective induction of necroptosis in apoptosis‐resistant cells, or the initiation of ferroptosis in cells laden with iron and undergoing oxidative stress, represents precision strategies in cancer treatment.^10^, ^18^ Furthermore, the role of cell death extends beyond the mere elimination of cancer cells, it also shapes the immune response against tumors.^19^ For example, the release of damage‐associated molecular patterns (DAMPs) during necroptosis can enhance antitumor immunity, suggesting a dual benefit of targeting cell death pathways.^20^, ^21^ All in all, recent studies have revealed that these pathways are not only pivotal for tumor suppression but also offer new therapeutic opportunities. Cancer cells often adapt to avoid traditional apoptosis, necessitating research into alternative cell death pathways that can potentially remove these resistant cells.

Therefore, the review aims to provide a comprehensive analysis of principal cell death pathways implicated in cancer, detailing their molecular characteristics, regulation, and significance in cancer therapeutics. By dissecting the specifics of each pathway, including apoptosis, necroptosis, autophagy, ferroptosis, and pyroptosis, this study will illuminate their unique impacts in oncology and emerging therapeutic angles. We intend to scrutinize key regulatory proteins in these pathways, evaluate current drugs targeting these mechanisms, and discuss ongoing clinical trials and associated challenges such as drug resistance. Special attention will be given to the modulation of these pathways by the TME and significant signaling cascades like phosphoinositide 3‐kinase (PI3K)/protein kinase B (AKT)/mechanistic target of rapamycin (mTOR), mitogen‐activated protein kinase (MAPK)/extracellular signal‐regulated kinase (ERK), and nuclear factor‐kappaB (NF‐κB).

In conclusion, this review promises to provide a comprehensive overview of cell death mechanisms in cancer, from molecular underpinnings to therapeutic applications, offering insights into the development of more effective and selective cancer therapies. It is intended not only to guide current therapeutic approaches but also to spur further research and innovation in personalized medicine, potentially revolutionizing cancer treatment strategies. Through meticulous analysis and a forward‐looking perspective, we aim to contribute significantly to the field of cancer research and therapy.

## 2 TYPES OF CELL DEATH PATHWAYS

### 2.1 Apoptosis

**FIGURE 1:**
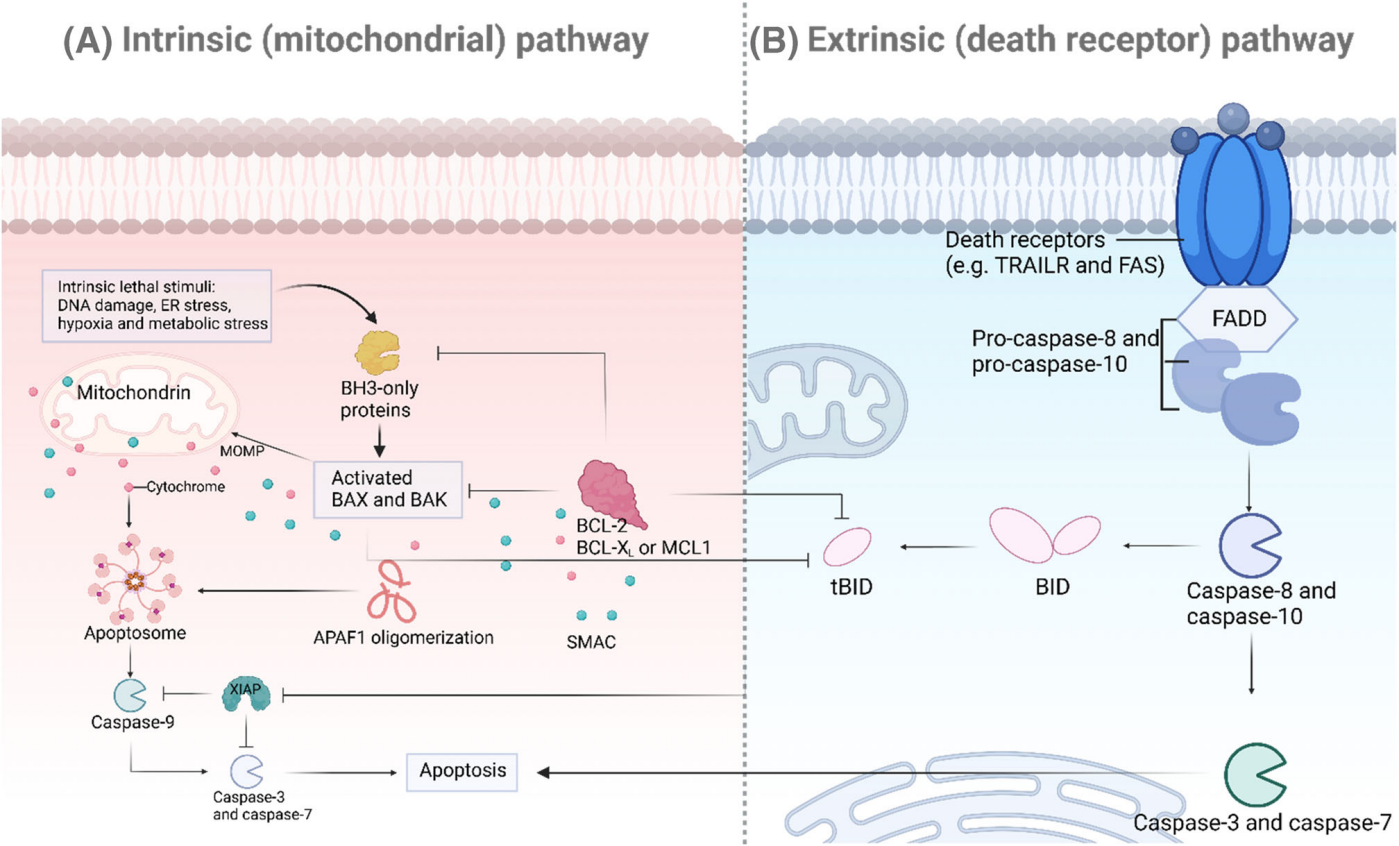
Schematic overview of apoptotic pathways. (A) Intrinsic pathway of apoptosis: Stress signals such as DNA damage or growth factor deprivation induce the activation of proapoptotic BH3‐only proteins (e.g., Bid, Bim, Puma) that activate Bax and Bak. These proteins oligomerize to form pores in the mitochondrial outer membrane, leading to the release of cytochrome *c* into the cytosol. Cytochrome *c* then binds to Apaf‐1 and ATP, resulting in the formation of the apoptosome complex that activates procaspase‐9. Activated caspase‐9 cleaves and activates downstream effector caspases (caspase‐3, caspase‐7), culminating in cell dismantling and apoptosis. (B) Extrinsic pathway of apoptosis: Binding of death ligands (e.g., FasL, TRAIL) to their respective death receptors (e.g., Fas, DR4, DR5) on the cell surface promotes receptor trimerization and the recruitment of the adaptor protein FADD. FADD attracts procaspase‐8, leading to the formation of the death‐inducing signaling complex (DISC). BAK, BCL2 antagonist/killer; BAX, Bcl‐2 associated X protein; Bcl‐2, B‐cell lymphoma‐2; BCL‐XL, B‐cell lymphoma‐extra large; FAS, soluble Fas ligand; FADD, Fas‐associating protein with a novel death domain; MCL1, myeloid cell leukemia‐1; TRAIL, TNF‐related apoptosis‐inducing ligand.

Apoptosis, a programmed cell death process, is crucial for maintaining cellular balance and development.^22^ Two main pathways, the intrinsic (mitochondrial) and the extrinsic (death receptor) pathways, regulate this process (Figure 1).^23^ This portion will examine the molecular mechanisms of these pathways, emphasizing the roles of Bcl‐2 family proteins, cytochrome *c* release, and caspase activation in the intrinsic pathway, along with the participation of death receptors and caspase‐8 activation in the extrinsic pathway.

#### 2.1.1 Intrinsic (mitochondrial) pathway

The intrinsic pathway of apoptosis is primarily regulated by mitochondrial signals and is critically dependent on the balance between proapoptotic and antiapoptotic members of the Bcl‐2 family of proteins (Figure 1A). This pathway is typically triggered by various forms of cellular stress, including DNA damage, oxidative stress, and growth factor deprivation.^24^, ^25^

##### Role of Bcl‐2 family proteins

The Bcl‐2 family of proteins is crucial in controlling the intrinsic pathway, with both proapoptotic and antiapoptotic members determining cell fate.^26^, ^27^ Antiapoptotic proteins like Bcl‐2 and Bcl‐xL maintain mitochondrial function by blocking proapoptotic proteins.^28^ Proapoptotic proteins like Bax, Bak, Bid, Bim, and Puma are activated by BH3‐only proteins upon apoptotic stimuli, leading to the oligomerization and permeabilization of the outer mitochondrial membrane.^29^, ^30^, ^31^

##### Cytochrome *c* release and caspase activation

The release of mitochondrial intermembrane space proteins, including cytochrome *c*, occurs when Bax and Bak permeabilize the outer membrane of the mitochondria.^32^

Cytochrome *c* then binds to apoptotic protease activating factor‐1 (Apaf‐1) and adenosine triphosphate (ATP) in the cytosol, which helps in the formation of the apoptosome. This complex recruit and activates procaspase‐9, leading to the activation of downstream effector caspases like caspase‐3 and caspase‐7, ultimately causing apoptosis by cleaving cellular substrates.^33^, ^34^, ^35^

#### 2.1.2 Extrinsic (death receptor) pathway

The initiation of the extrinsic pathway occurs when extracellular death ligands bind to their respective death receptors on the cell surface (Figure 1B), a process essential for the immune system in eliminating infected or transformed cells.^36^, ^37^, ^38^

##### Death receptors (e.g., Fas, TRAIL)

Members of the tumor necrosis factor (TNF) receptor superfamily are death receptors, which consist of receptors like Fas (CD95) and TNF‐related apoptosis‐inducing ligand (TRAIL) receptors (DR4 and DR5). These receptors have a crucial intracellular death domain (DD) that is necessary for transmitting signals that lead to apoptosis. When death ligands (such as FasL and TRAIL) bind to their respective receptors, it causes receptor trimerization and the recruitment of adaptor proteins such as Fas‐associated protein with death domain (FADD).^39^, ^40^

##### Caspase‐8 activation

Recruiting FADD to the death receptor complex helps facilitate the binding of procaspase‐8 via its death effector domain, forming the death‐inducing signaling complex (DISC). Within the DISC, procaspase‐8 molecules are brought close together, leading to their dimerization and activation. Once activated, caspase‐8 can directly cleave and activate downstream effector caspases, like caspase‐3, to start the apoptotic process. Furthermore, caspase‐8 can also cleave the BH3‐only protein Bid, generating truncated Bid, which moves to mitochondria to enhance apoptotic signaling through the intrinsic pathway.^41^, ^42^, ^43^

In summary, the intrinsic and extrinsic pathways of apoptosis are intricate and closely controlled mechanisms crucial for upholding cellular integrity and overall organismal well‐being. Studying the molecular details of these pathways provides valuable information for developing therapeutic approaches for conditions marked by perturbed apoptosis, including cancer.

### 2.2 Necrosis and necroptosis

Cell death is a fundamental biological process that ensures the removal of damaged or unnecessary cells.^44^ While apoptosis has been extensively studied, necrosis and its regulated form, necroptosis, have gained increasing attention, particularly in the context of disease pathogenesis.^45^ This section will delineate the characteristics of necrosis and necroptosis, emphasizing the molecular mechanisms and the roles of key players such as receptor‐interacting protein kinase 1 (RIPK1), receptor‐interacting protein kinase 3 (RIPK3), and mixed lineage kinase domain‐like protein (MLKL).^46^, ^47^

#### 2.2.1 Characteristics of necrosis

Necrosis is typically seen as a type of cell death that occurs when there is sudden damage to the cells. ^24^ This kind of cell death is identified by a breakdown in membrane integrity, uncontrolled release of cell contents, and inflammation that follows.^46^ Unlike apoptosis, necrosis does not involve specific signaling pathways, and it typically results from factors such as trauma, infection, or ischemia. Morphologically, necrotic cells exhibit swelling (oncosis), organelle disruption, and eventual rupture of the plasma membrane. The release of intracellular components into the extracellular space due to these events triggers an inflammatory response from the immune system, which can lead to tissue damage and disease progression because of the uncontrolled nature of necrosis and the associated inflammation.^48^

#### 2.2.2 Regulated necrosis: necroptosis

**FIGURE 2:**
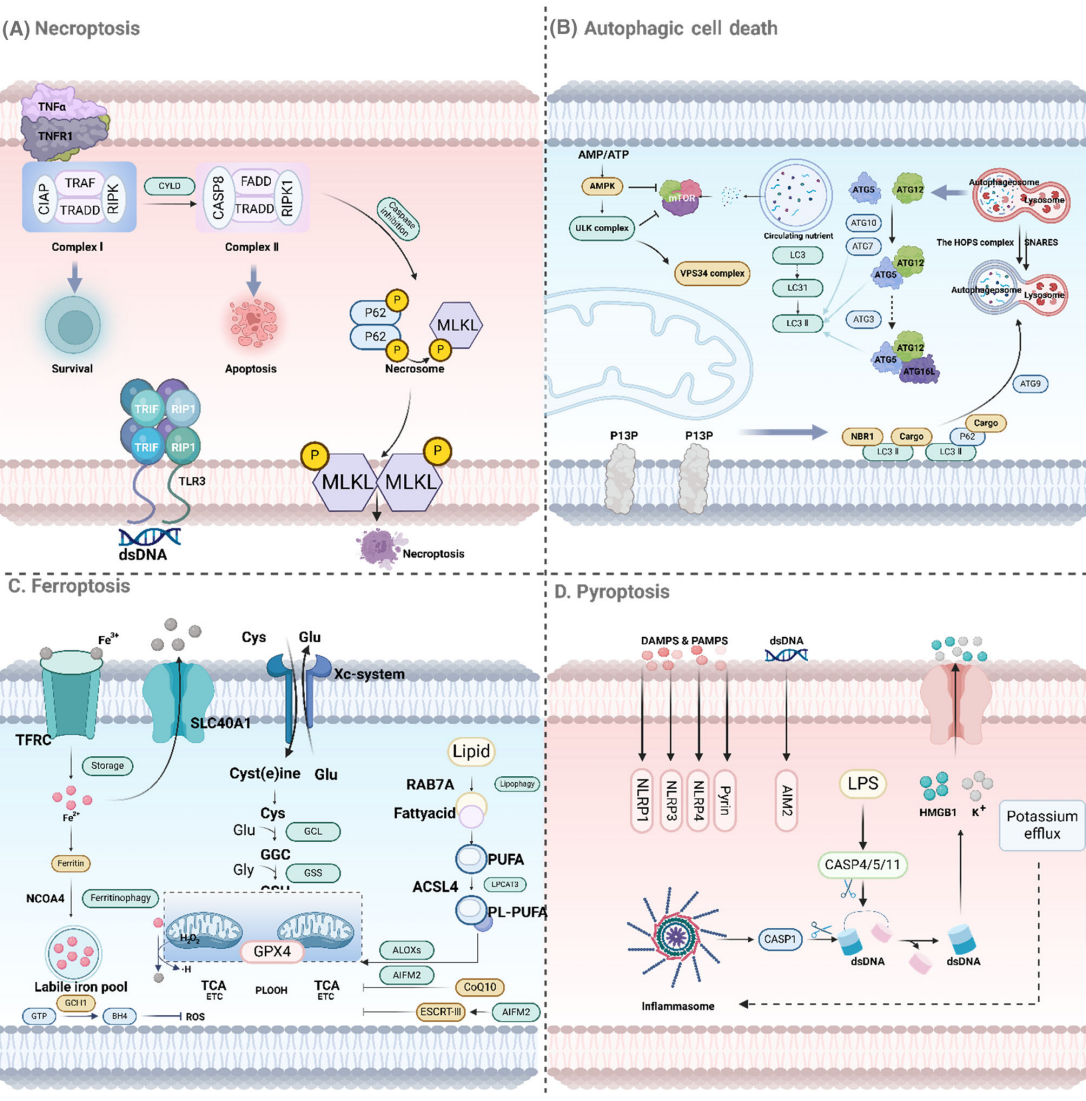
Molecular pathways and cellular mechanisms of different forms of cell death. (A) Necrosis is characterized by the loss of plasma membrane integrity and release of cellular contents, leading to inflammation. Morphologically, necrotic cells show cell and organelle swelling, and membrane rupture. Necroptosis involves the regulated activation of RIPK1, RIPK3, and MLKL following the triggering by death receptors like TNFR1. RIPK1 forms a complex with RIPK3, leading to RIPK3 activation and subsequent phosphorylation of MLKL, which then translocates to the plasma membrane to form disruptive pores. (B) Autophagy proceeds through stages of initiation by the ULK1 complex, nucleation of the phagophore mediated by the class III PI3K complex, elongation involving two ubiquitin‐like systems, and ultimately maturation into an autolysosome where contents are degraded and recycled. (C) Ferroptosis is driven by iron‐dependent lipid peroxidation. Iron uptake via transferrin receptors and its storage or availability in the labile iron pool enables the Fenton reaction, leading to lipid peroxidation at cellular membranes. Regulatory mechanisms involve the antioxidative actions of GPX4 and the cystine/glutamate antiporter system Xc⁻. (D) Pyroptosis features the activation of inflammatory caspases and the formation of gasdermin D pores in the plasma membrane. Activation of caspase‐1, ‐4, ‐5, and ‐11, leads to the cleavage of GSDMD, and the N‐terminal pore‐forming fragment of GSDMD induces the formation of membrane pores, resulting in cell swelling, lysis, and release of inflammatory cytokines. ACSL4, acyl‐CoA synthetase long chain family member 4; AMP, antimicrobial peptides; ATP, adenosine triphosphate; AMPK, adenosine 5′‐monophosphate (AMP)‐activated protein kinase; ALOXs, arachidonic acid lipoxygenase; AIFM2, ferroptosis suppressor protein 1; ATG5, autophagy‐related gene 5; ATG7, autophagy‐related gene 7; ATG9, autophagy‐related gene 9; ATG10, autophagy‐related gene 10; ATG12, autophagy‐related gene 12; CIAP, Cellular inhibitor of apoptosis; FADD, Fas‐associating protein with a novel death domain; GCH1, guanosine triposphate cyclohydrolase1; GPX4, glutathione peroxidase 4; LPS, lipopolysaccharides; MLKL, mixed lineage kinase domain‐like protein; NCOA4, nuclear receptor coactivator 4; NLRP1, NLR family pyrin domain containing 1; NLRP3, NLR family pyrin domain containing 3; NLRP4, NLR family pyrin domain containing 4; P62, sequestosome; PUFA, polyunsaturated fatty acids; RIPK, receptor‐interacting protein kinase 1; RIP1, receptor‐interacting protein 1; RAB7A, RAS‐related in brain 7A; SLC40A1, solute carrier family 40 member 1; TNFα, tumor necrosis factor‐α; TNFR1, tumor necrosis factor receptor 1; TRAF, tumor necrosis factor receptor‐associated factors; TRADD, TNFR1‐associated death domain protein; TFRC, transferrin receptor.

Necroptosis is a form of programmed necrosis that shares morphological features with necrosis but is executed through regulated signaling pathways. This process can be triggered under conditions where apoptosis is inhibited, serving as a backup mechanism to ensure cell death.^49^, ^50^, ^51^ Necroptosis plays a crucial role in various physiological and pathological contexts, including development, immune response, and disease.^52^, ^53^, ^54^ The molecular machinery governing necroptosis involves a well‐coordinated interaction between RIPKs and MLKL.^55^ The key steps in the necroptotic pathway are as follows: ① *RIPK1 activation*: Necroptosis is often initiated by the activation of death receptors, such as TNF receptor 1 (TNFR1), which can recruit RIPK1 through its DD. Upon activation, RIPK1 undergoes autophosphorylation and forms a complex with RIPK3, known as the necrosome.^56^, ^57^ ② *RIPK3 activation*: Within the necrosome, RIPK3 is phosphorylated by RIPK1. This phosphorylation event is critical for the downstream signaling cascade that leads to necroptosis. RIPK3, in turn, recruits and phosphorylates MLKL.^58^, ^59^ ③ *MLKL activation and membrane disruption*: Phosphorylated MLKL undergoes a conformational change, allowing it to oligomerize and translocate to the plasma membrane. Once at the membrane, MLKL disrupts membrane integrity by forming pores, leading to cell swelling, membrane rupture, and ultimately necrotic cell death (Figure 2A).^60^

To sum up, necrosis and necroptosis are two different but related types of cell death. Whereas necrosis occurs from sudden damage to cells, necroptosis is a controlled form of cell death involving RIPK1, RIPK3, and MLKL.^61^, ^62^ Understanding the molecular mechanisms behind these processes can help us better comprehend their impact on health and disease, and potentially identify new treatment targets for disorders marked by abnormal cell death.

### 2.3 Autophagic cell death

Autophagy is an essential cellular process that is responsible for breaking down and recycling components of the cytoplasm. Although it is mainly a survival mechanism, autophagy can sometimes result in a type of programmed cell demise referred to as autophagic cell death.^63^, ^64^ This segment will delve into the mechanisms of autophagy and its dual function in cancer.

#### 2.3.1 Mechanisms of autophagy

Autophagy, a catabolic process involving the breakdown of cellular components through lysosomal machinery, is derived from the Greek words “self” and “eating.”^65^ The process can be divided into several key stages: ① *Initiation*: Autophagy starts with the activation of the ULK1 complex, which consists of ULK1, FIP200, ATG13, and ATG101, and is regulated by nutrient and energy sensors like mTOR and AMP‐activated protein kinase (AMPK).^66^ ② *Nucleation*: The activated ULK1 complex triggers the formation of the phagophore, a double‐membraned structure, with the help of the class III PI3K complex containing VPS34, Beclin‐1, and ATG14L, creating phosphatidylinositol 3‐phosphate (PI3P) to recruit other autophagy‐related proteins.^66^ ③ *Elongation and maturation*: The phagophore expands to engulf cytoplasmic material, with the assistance of the ATG12–ATG5–ATG16L complex and the LC3–PE conjugation system. LC3‐II is crucial for membrane expansion and cargo recognition.^67^ ④ The autophagosome merges with lysosomes to form an autolysosome, where lysosomal hydrolases break down the sequestered material into basic biomolecules that can be recycled for reuse in the cytoplasm (Figure 2B).^68^

#### 2.3.2 Role of autophagy in cancer

Autophagy plays a complex and context‐dependent role in cancer, acting as a double‐edged sword with both tumor‐suppressive and tumor‐promoting effects.^69^ In the early stages of cancer development, autophagy can act as a tumor suppressor.^70^ By degrading damaged organelles and proteins, autophagy prevents the accumulation of cellular damage and maintains genomic stability. This process reduces oxidative stress and inhibits chronic inflammation, both of which are associated with oncogenesis. Furthermore, autophagy can induce autophagic cell death in cancer cells, especially under conditions of metabolic stress or treatment with chemotherapeutic agents.^69^, ^71^ Paradoxically, in established tumors, autophagy often supports cancer cell survival and growth. Tumor cells exploit autophagy to withstand metabolic stress, hypoxia, and nutrient deprivation commonly found in the TME. By recycling intracellular components, autophagy provides essential substrates for energy production and biosynthesis, promoting tumor cell proliferation and survival. Additionally, autophagy can contribute to therapy resistance by enabling cancer cells to survive cytotoxic treatments.^72^, ^73^ Given its dual role, targeting autophagy in cancer therapy requires a nuanced approach. Inhibiting autophagy may be beneficial in established tumors where autophagy supports survival and growth. Several clinical trials are investigating autophagy inhibitors, such as hydroxychloroquine (HCQ), in combination with conventional therapies.^74^ Conversely, enhancing autophagy could be a strategy to induce autophagic cell death in certain cancer types or sensitize tumors to chemotherapy and radiation.^75^

In conclusion, autophagy is a multifaceted process that plays critical roles in cellular homeostasis and disease. Its involvement in cancer is particularly complex, encompassing both tumor‐suppressive and tumor‐promoting functions.^76^ Understanding the context‐dependent nature of autophagy in cancer is essential for developing effective therapeutic strategies that harness or inhibit this process to benefit patients.

### 2.4 Ferroptosis

Ferroptosis is a unique type of controlled cell death that involves iron‐dependent lipid peroxidation.^77^ This process is distinct in its morphology, biochemistry, and genetics compared with other forms of cell death like apoptosis, necrosis, and autophagy.^12^, ^78^ Recent years have seen a significant advancement in the understanding of ferroptosis, highlighting its crucial functions in different physiological and pathological situations, including cancer.^79^, ^80^

#### 2.4.1 Iron‐dependent lipid peroxidation

The accumulation of lipid peroxides drives ferroptosis, which can become toxic when levels are excessive, and is reliant on iron, a transition metal that triggers the production of reactive oxygen species (ROS) via the Fenton reaction.^81^, ^82^ The key steps in iron‐dependent lipid peroxidation include iron uptake and storage, ROS generation, and lipid peroxidation.^83^ Iron is taken up by cells via transferrin receptor‐mediated endocytosis of transferrin‐bound iron, which is then released from endosomes and stored in the cytosol in a labile iron pool or sequestered in ferritin. Free iron in the labile iron pool can participate in the Fenton reaction, converting hydrogen peroxide (H₂O₂) into highly reactive hydroxyl radicals (•OH), which initiate lipid peroxidation by abstracting hydrogen atoms from polyunsaturated fatty acids in membrane phospholipids. The initial lipid radicals formed are further oxidized to lipid peroxides, which, if not adequately detoxified, can decompose into reactive aldehydes and propagate further lipid peroxidation, leading to the loss of membrane integrity and cell death.^84^, ^85^, ^86^

#### 2.4.2 Regulation by GPX4 and system Xc^−^

Critical antioxidant systems are involved in regulating ferroptosis to prevent the buildup of lipid peroxides, with two main regulators being glutathione peroxidase 4 (GPX4) and the cystine/glutamate antiporter system Xc⁻.^87^ GPX4, a selenoprotein, is essential in protecting cells from ferroptosis by converting lipid hydroperoxides (LOOH) into alcohols, thus stopping lipid peroxidation.^88^, ^89^ GPX4 requires glutathione (GSH) as a substrate to exert its peroxidase activity. It specifically reduces LOOH within membranes to nontoxic lipid alcohols, thus inhibiting the chain reaction of lipid peroxidation. The activity of GPX4 is directly linked to the intracellular levels of GSH, which in turn is synthesized from cysteine. Therefore, the availability of cysteine is crucial for maintaining GPX4 activity and preventing ferroptosis.^90^ System Xc⁻ is a plasma membrane transporter composed of two subunits, SLC7A11 and SLC3A2, which mediates the exchange of extracellular cystine and intracellular glutamate. By importing cystine into the cell in exchange for glutamate, system Xc⁻ facilitates the synthesis of GSH. Cystine is reduced to cysteine inside the cell, which is then used to synthesize GSH. The activity of system Xc⁻ is influenced by various factors, including oxidative stress and nutrient availability. ^91^, ^92^

In summary, ferroptosis is a controlled form of cell death triggered by iron‐induced lipid peroxidation. The susceptibility of cells to ferroptosis depends on the balance between the production of lipid peroxides and their removal by antioxidant mechanisms like GPX4 and system Xc⁻ (Figure 2C). Understanding the molecular mechanisms underlying ferroptosis provides valuable insights into its role in various diseases and offers potential therapeutic targets for conditions where ferroptosis plays a critical role.

### 2.5 Pyroptosis

Pyroptosis is a unique form of programmed cell death that differs from apoptosis and necrosis, known for its inflammatory characteristics.^93^ It is usually induced by infections and various diseases, resulting in the activation of inflammatory caspases and the creation of gasdermin pores in the cell membrane.^94^, ^95^ Here, we will delve into the key mechanisms involved in pyroptosis, focusing on the role of inflammatory caspases and gasdermin proteins.

#### 2.5.1 Inflammatory caspases

Inflammatory caspases, including caspase‐1, ‐4, ‐5, and ‐11, are crucial for initiating and carrying out pyroptosis.^96^, ^97^ Caspase‐1 is activated by pathogen‐associated molecular patterns and DAMPs through inflammasomes like the NLRP3 inflammasome.^98^, ^99^ Once activated, it converts pro‐IL‐1β and pro‐IL‐18 into IL‐1β and IL‐18, and triggers pyroptosis by cleaving GSDMD.^100^ On the other hand, caspase‐4, ‐5, and ‐11 are activated by intracellular lipopolysaccharides without an inflammasome, also leading to pyroptosis by cleaving GSDMD. While they do not directly process pro‐IL‐1β and pro‐IL‐18, their activation can enhance the inflammatory response through secondary pathways.^101^, ^102^

#### 2.5.2 Formation of gasdermin pores

The main mechanism behind pyroptosis involves GSDMD, a protein from the gasdermin family, creating pores in the cell membrane.^103^ When activated, inflammatory caspases break down GSDMD at a specific location, separating its pore‐forming domain from its inhibitory domain. This separation releases the pore‐forming domain, which moves to the cell membrane and binds to phospholipids. Multiple pore‐forming domains then come together to create large pores, disrupting the cell membrane and causing cell swelling, membrane rupture, and the release of cell contents. These pores allow proinflammatory cytokines like IL‐1β and IL‐18 to be released, promoting inflammation by attracting and activating immune cells. Ultimately, the cell membrane ruptures, releasing DAMPs and amplifying the immune response to further contribute to inflammation.^96^, ^104^, ^105^

In summary, pyroptosis is a type of cell death that involves inflammation, triggered by activating inflammatory caspases and creating gasdermin pores. Inflammatory caspases like caspase‐1, ‐4, ‐5, and ‐11 are essential for detecting harmful signals, causing the breakdown of GSDMD. The GSDMD N‐terminal pieces generate pores in the cell membrane, leading to cell rupture and the discharge of proinflammatory substances (Figure 2D). Understanding the mechanisms of pyroptosis provides insights into its roles in various diseases, particularly those involving infection and inflammation, and offers potential targets for therapeutic intervention.

## 3 MOLECULAR MECHANISMS REGULATING CELL DEATH

The regulation of cell death pathways is intricately controlled by both genetic and epigenetic factors.^106^ Genetic mutations and epigenetic modifications can significantly influence the susceptibility of cells to various forms of cell death, including apoptosis, necrosis, ferroptosis, and pyroptosis.^107^ Here, we will focus on the impact of mutations in key regulators such as tumor protein p53 (TP53) and Bcl‐2 family genes, as well as epigenetic modifications like DNA methylation and histone modification.

### 3.1 Genetic and epigenetic factors

#### 3.1.1 Mutations in key regulators

##### Tumor protein p53

TP53 is an essential gene that produces the p53 protein, which is responsible for maintaining genomic stability by controlling the cell cycle, DNA repair, and apoptosis.^108^ Mutations in TP53 are frequently seen in human cancers and can cause the loss of p53's ability to suppress tumors, resulting in uncontrolled cell growth and resistance to cell death. These mutations prevent p53 from activating genes that promote apoptosis, such as BAX, PUMA, and NOXA, leading to inhibition of cell death. Some TP53 mutations can even give the protein oncogenic characteristics, supporting cell survival, and growth.^109^

##### Bcl‐2 family genes

The intrinsic pathway of apoptosis is regulated by the Bcl‐2 family of proteins, which includes both proapoptotic (such as BAX, BAK) and antiapoptotic members (like Bcl‐2, Bcl‐xL).^110^ Changes in the expression or function of these genes can disrupt the balance between cell survival and death. In many cancers, increased levels of antiapoptotic proteins such as Bcl‐2 and Bcl‐xL prevent the activation of proapoptotic proteins, promoting cell survival and resistance to apoptotic signals. On the other hand, mutations or deletions in proapoptotic genes such as BAX or BAK can hinder the apoptotic process, leading to tumorigenesis.^111^, ^112^

#### 3.1.2 Epigenetic modifications

##### DNA methylation

DNA methylation involves adding a methyl group to cytosine residues at the 5‐position, mainly in CpG dinucleotides, which affects gene expression by changing DNA accessibility to transcription factors. In terms of cell death, hypermethylation of tumor suppressor gene promoters, such as P16 and DAPK, can silence them and aid in cancer progression by blocking apoptosis. Conversely, global DNA hypomethylation can result in genomic instability and the activation of oncogenes, further promoting cell survival and proliferation.^113^, ^114^, ^115^

##### Histone modification

Histone proteins, wrapped around DNA, can be modified after translation in various ways, such as methylation, acetylation, phosphorylation, and ubiquitination, which can affect chromatin structure and gene expression. In relation to cell death, histone acetylation usually promotes the activation of proapoptotic genes, while deacetylation by histone deacetylases can suppress these genes, preventing apoptosis.^116^ Similarly, histone methylation can either enhance or inhibit gene expression, depending on the specific residues impacted; for example, trimethylation of histone H3 lysine 4 (H3K4me3) is connected to gene activation, while trimethylation of histone H3 lysine 27 (H3K27me3) is associated with gene repression. Alterations in these histone modifications can have a significant impact on the regulation of genes involved in cell death processes.^116^, ^117^

**FIGURE 3:**
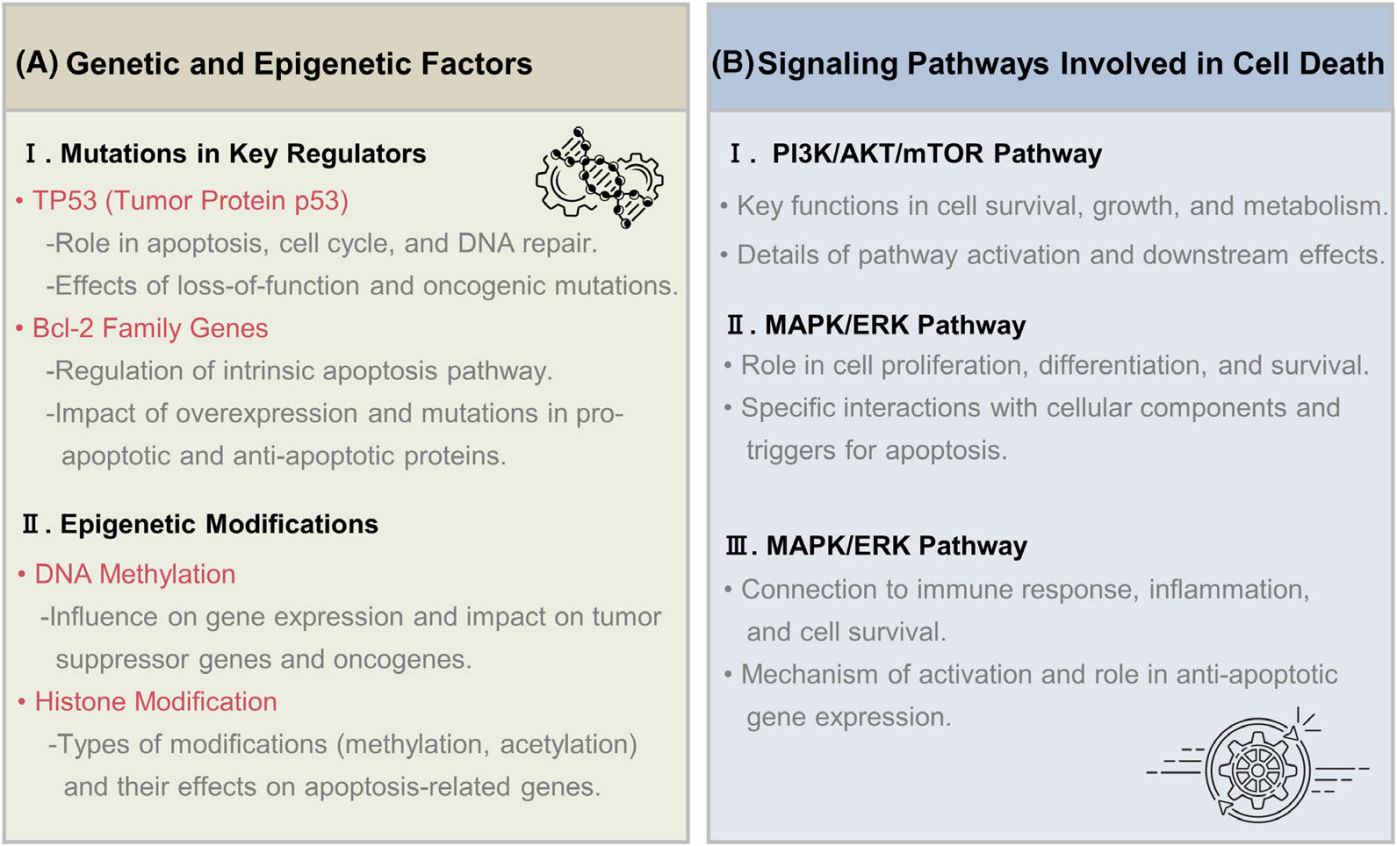
Overview of the regulatory mechanisms and signaling pathways involved in cell death and survival. (A) This panel illustrates the role of mutations in genes such as TP53 and members of the Bcl‐2 family, highlighting how these alterations can disrupt the balance between cell survival and apoptosis. The diagram emphasizes the impact of TP53 mutations on the suppression of proapoptotic genes and the resulting evasion of apoptosis. Additionally, epigenetic influences such as DNA methylation and histone modifications are shown modulating gene expression related to apoptosis, with examples including hypermethylation of P16 and the effects of histone acetylation on chromatin structure and gene activity. (B) This panel maps out three crucial signaling pathways: PI3K/AKT/mTOR, MAPK/ERK, and NF‐κB, each depicted with their main components and interactions. The schematic shows how growth factors activate PI3K leading to AKT and mTOR signaling cascades that promote cell survival and growth. The MAPK/ERK pathway is initiated by activated RTKs leading to downstream activation of transcription factors that drive proliferation and survival. The NF‐κB pathway's activation via IKK complex and subsequent nuclear translocation of NF‐κB, resulting in the transcription of genes that prevent apoptosis and foster cell proliferation, is also delineated. AKT, Protein kinase B; Bcl‐2, B‐cell lymphoma‐2; ERK, extracellular regulated protein kinases; MAPK, mitogen activated protein kinase; mTOR, mammalian target of rapamycin; NF‐κB, nuclear factor kappa‐light‐chain‐enhancer of activated B cells; PI3K, phosphoinositide 3‐kinase.

In summary, both genetic and epigenetic factors play crucial roles in regulating cell death. Mutations in key regulators such as TP53 and Bcl‐2 family genes can disrupt normal cell death mechanisms, contributing to diseases like cancer. Epigenetic modifications, including DNA methylation and histone modifications, further modulate the expression of genes involved in cell death, influencing cellular responses to stress and damage (Figure 3A). Having a grasp on these genetic and epigenetic factors gives us valuable information about how cell death works and presents possible targets for treating different diseases.

### 3.2 Signaling pathways involved

Cell death regulation is governed by a variety of signaling pathways that either promote cell survival or facilitate cell death in response to various stimuli. Key signaling pathways involved in these processes include the PI3K/AKT/mTOR pathway, the MAPK/ERK pathway, and NF‐κB signaling (Figure 3B). Each of these pathways plays a critical role in determining cell fate by integrating signals from the cellular environment and modulating the activity of various downstream effectors.

#### 3.2.1 PI3K/AKT/mTOR pathway

The PI3K/AKT/mTOR pathway plays a crucial role in regulating cell survival, growth, proliferation, and metabolism, with its disruption being common in cancer and other diseases.^118^ Growth factors, cytokines, and other external signals can activate PI3K through receptor tyrosine kinases (RTKs) or G protein‐coupled receptors. Upon activation, PI3K phosphorylates phosphatidylinositol (4,5)‐bisphosphate (PIP2) to produce phosphatidylinositol (3,4,5)‐trisphosphate (PIP3), a lipid second messenger. PIP3 then recruits AKT to the cell membrane, where it is phosphorylated and activated by phosphoinositide‐dependent kinase‐1 and mTOR complex 2 (mTORC2). Once activated, AKT supports cell survival and growth by phosphorylating and inhibiting proapoptotic substances like BAD and FOXO, while also enhancing the activity of prosurvival proteins such as Murine double minute 2. mTOR, a serine/threonine kinase, operates in two different complexes known as mTORC1 and mTORC2. AKT directly activates mTORC1 by inhibiting the TSC1/TSC2 complex, which negatively regulates mTORC1. mTORC1 promotes protein synthesis, cell growth, and survival by phosphorylating downstream targets like S6K1 and 4E‐BP1, while mTORC2 phosphorylates AKT, further enhancing its activity.^119^, ^120^, ^121^

#### 3.2.2 MAPK/ERK pathway

The MAPK/ERK pathway plays a crucial role in controlling cell proliferation, differentiation, and survival, with key components including rat sarcoma (RAS), rapidly accelerated fibrosarcoma (RAF), mitogen‐activated protein kinase kinase (MEK), and ERK. Activation occurs when external signals stimulate RTKs, leading to RAS activation and subsequent recruitment of RAF kinases. RAF then activates MEK, which in turn activates ERK. ERK travels to the nucleus and activates transcription factors like ELK1 and c‐FOS, resulting in gene expression related to cell growth and survival. ERK also contributes to cell survival by inhibiting proapoptotic proteins like BAD and increasing the levels of antiapoptotic proteins such as Bcl‐2 and MCL‐1. Nevertheless, prolonged activation of the MAPK/ERK pathway under stress conditions can trigger cell death by activating proapoptotic genes.^122^, ^123^

#### 3.2.3 NF‐κB signaling

The NF‐κB pathway plays a crucial role in regulating immune responses, inflammation, and cell survival.^124^ The NF‐κB family consists of transcription factors like p65 (RelA), p50, and c‐Rel, which combine to form different homo‐ and heterodimers. Activation of NF‐κB can occur in response to various stimuli, such as proinflammatory cytokines like TNF‐α and IL‐1, microbial products, and stress signals. These stimuli trigger the IκB kinase (IKK) complex to phosphorylate and break down IκB proteins that inhibit NF‐κB. The breakdown of IκB enables NF‐κB dimers to move into the nucleus. In the nucleus, NF‐κB binds to specific DNA sequences and activates the transcription of genes associated with inflammation, immune response, cell survival, and cell proliferation.^125^, ^126^ This pathway promotes cell survival by elevating the expression of antiapoptotic genes like Bcl‐2, Bcl‐xL, and x‐linked inhibitor of apoptosis protein, as well as genes involved in cell proliferation such as c‐MYC. Furthermore, NF‐κB activation can lead to the production of proinflammatory cytokines and chemokines, which can either support cell survival or contribute to cell death during chronic inflammation.^124^, ^127^, ^128^

In essence, the PI3K/AKT/mTOR, MAPK/ERK, and NF‐κB signaling pathways play a vital role in controlling cell fate determinations by incorporating external signals to regulate the functions of different proteins connected to cell survival and apoptosis. Disorders in these pathways are frequently linked to illnesses like cancer, underscoring their significance as possible treatment targets. Comprehending the complexities of these signaling systems offers valuable knowledge about the processes that oversee cell survival and death.

### 3.3 Role of the TME

**FIGURE 4:**
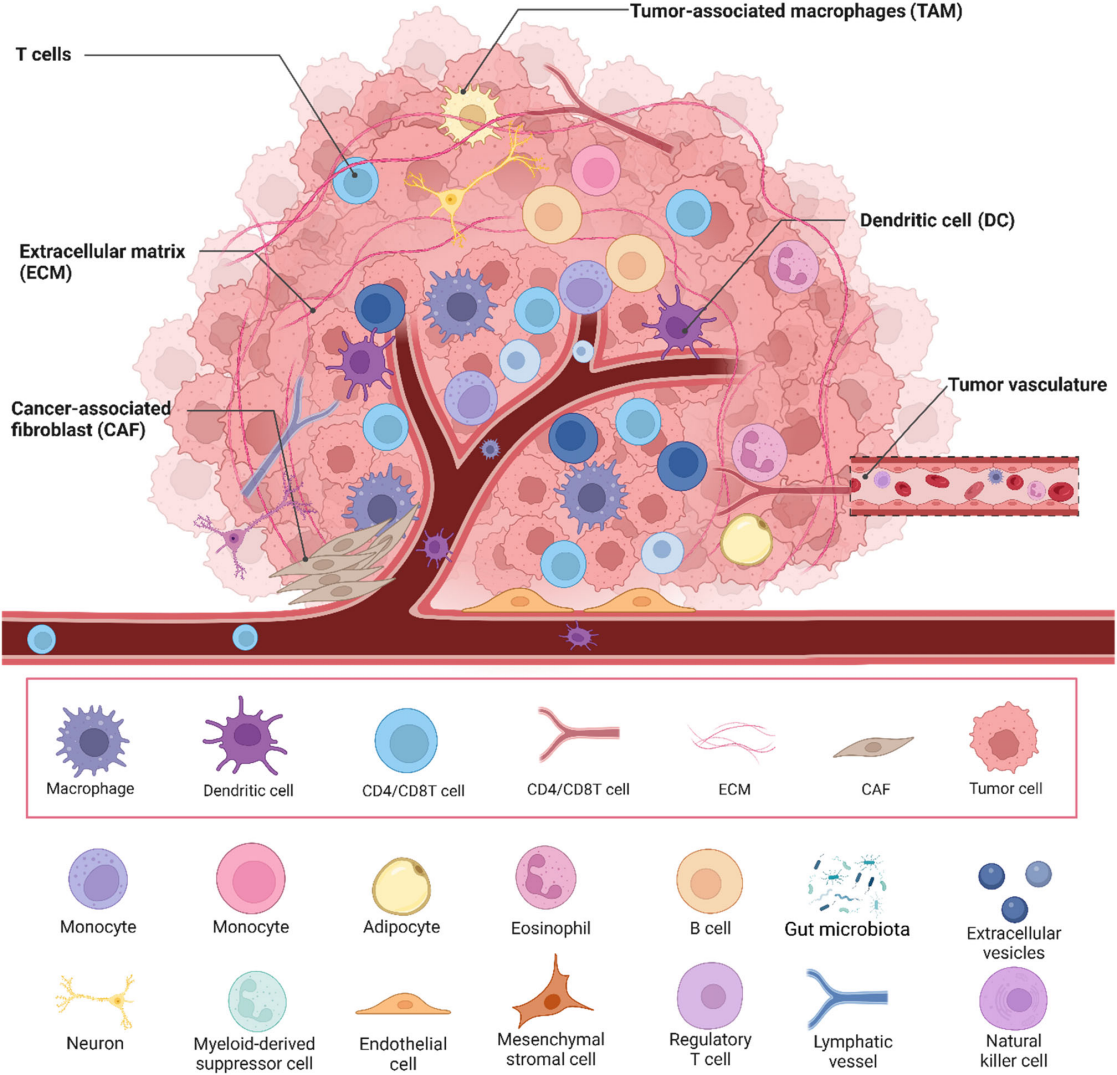
Schematic representation of the tumor microenvironment (TME). The figure illustrates the complex network within the TME, the interaction between cancer cells, immune cells (such as TAM, T cells), and stromal cells, including cancer‐associated fibroblasts (CAF) and endothelial cells, is shown. The figure also highlights the immunosuppressive environment facilitated by various cellular interactions that promote tumor progression and evasion from immune surveillance.

The TME is essential for tumor progression, metastasis, and treatment effectiveness, as it consists of a mix of cell types and molecules, such as cancer cells, immune cells, stromal cells, blood vessels, ECM, and signaling molecules (Figure 4).^125^, ^129^ Understanding the TME is essential for comprehending tumor biology and developing effective cancer therapies. Key aspects of the TME include hypoxia and nutrient deprivation, as well as interactions with immune cells and stromal components.

#### 3.3.1 Hypoxia and nutrient deprivation

Hypoxia (low oxygen levels) and nutrient deprivation are common features of the TME due to the rapid growth of tumors outpacing their blood supply.^130^ As tumors grow, they often outstrip their blood supply, leading to regions with inadequate oxygen levels. This hypoxia stabilizes hypoxia‐inducible factors (HIF‐1α and HIF‐2α), which translocate to the nucleus and induce the expression of genes promoting angiogenesis (e.g., VEGF), glycolysis, and survival.^131^ Cancer cells adapt to hypoxia by shifting their metabolism towards anaerobic glycolysis (the Warburg effect), increasing glucose uptake and lactate production. Additionally, hypoxia can lead to resistance to radiotherapy and some chemotherapies, as low oxygen levels reduce the generation of ROS that mediate the cytotoxic effects of these treatments. Tumor cells also face nutrient deprivation, competing for limited resources such as glucose, amino acids, and lipids due to inadequate blood supply and high metabolic demand. In response, cancer cells often upregulate autophagy to recycle cellular components and maintain homeostasis. They also exhibit metabolic flexibility, using alternative substrates like fatty acids and glutamine to sustain growth and survival. Nutrient deprivation activates stress response pathways, such as the unfolded protein response and AMPK signaling, helping cells adapt to metabolic stress.^132^, ^133^, ^134^

#### 3.3.2 Interaction with immune cells and stromal components

The interaction between cancer cells and various components of the TME, including immune cells and stromal cells, significantly influences tumor progression and therapeutic response.^135^ Tumor‐associated macrophages in the TME often adopt an M2‐like phenotype, promoting tumor growth, angiogenesis, and immunosuppression through cytokines like IL‐10 and TGF‐β. Effective antitumor immune responses depend on cytotoxic T cells (CTLs), but tumors create an immunosuppressive environment that inhibits CTL function, while regulatory T cells accumulate and suppress effector T cells, aiding immune evasion.^136^ Myeloid‐derived suppressor cells (MDSCs) also contribute to immunosuppression by inhibiting T cell activation and promoting tumor immune evasion via arginase, nitric oxide, and ROS.^137^ Cancer‐associated fibroblasts (CAFs) secrete ECM components and matrix metalloproteinases (MMPs) to remodel the ECM, facilitating tumor invasion and metastasis, and produce growth factors and cytokines that promote tumor proliferation, survival, and angiogenesis. Endothelial cells respond to proangiogenic signals like VEGF from hypoxic tumor cells, forming new blood vessels that provide nutrients and oxygen to the tumor, though these vessels are often abnormal, leading to inefficient blood flow, increased permeability, and further hypoxia and nutrient deprivation. The ECM itself provides structural support and influences cell behavior through biochemical and biomechanical signals, while sequestering growth factors and cytokines, modulating their availability and activity within the TME.^138^, ^139^

In summary, the TME is a dynamic and complex network that profoundly influences tumor progression and response to therapy. Hypoxia and nutrient deprivation drive metabolic adaptations in cancer cells, while interactions with immune cells and stromal components create a supportive environment for tumor growth and immune evasion. Understanding these interactions is crucial for developing strategies to target the TME and improve cancer treatment outcomes.

## 4 THERAPEUTIC TARGETING OF CELL DEATH PATHWAYS IN CANCER

The induction of cell death in cancer cells is a fundamental strategy in cancer therapy.^140^ By understanding and manipulating various cell death pathways, we can develop targeted treatments that selectively kill cancer cells while minimizing damage to normal tissues. Here, we explore different therapeutic approaches targeting apoptosis, necroptosis, autophagy, ferroptosis, and pyroptosis (Table 1).

**TABLE 1:** The therapeutic agents targeting cancer cell death pathways: representative drugs, targets, and mechanisms.

Types	Representative drugs	Main targets	Main mechanisms	References
Apoptosis inducers	Venetoclax (ABT‐199)	Bcl‐2 family proteins	Inhibits Bcl‐2 to induce apoptosis in Bcl‐2‐dependent cells	141
	TRAIL, Agonistic antibodies targeting DR4 or DR5, Recombinant FasL	TRAIL receptors (DR4, DR5), Fas (CD95)	Activates death receptors leading to DISC formation, caspase‐8 activation, and initiation of apoptosis	142, 143, 144
Necroptosis modulators	Necrostatin‐1 (Nec‐1), GSK2982772	RIPK1	Blocks RIPK1 kinase activity, preventing necroptotic cascade initiation	145, 146, 147
	GSK‐872	RIPK	Inhibits RIPK3 kinase activity, preventing activation of MLKL	148, 149
	Necrosulfonamide	MLKL	Prevents oligomerization and membrane translocation of MLKL, blocking necroptosis execution phase	150
Autophagy modulators	Chloroquine (CQ), hydroxychloroquine (HCQ)	Autophagosomes and lysosomes	Inhibits lysosomal acidification, blocking fusion of autophagosomes with lysosomes	151, 152
Ferroptosis inducers	RSL3, ML210	GPX4	Inhibits GPX4, leading to accumulation of lipid hydroperoxides and triggering ferroptosis	153, 154
Ferroptosis modulators	Deferoxamine (DFO), deferiprone (DFP), liproxstatin‐1	Iron metabolism and lipid peroxidation	Modulates iron levels and lipid peroxidation to control ferroptosis	155
Pyroptosis inducers	Activators of caspase‐1, caspase‐4/5/11	Inflammatory caspases	Induces pyroptosis through activation of caspases leading to cell death and immune activation	156, 157

### 4.1 Apoptosis inducers

#### 4.1.1 BH3 mimetics targeting Bcl‐2 family proteins

Venetoclax (ABT‐199) is a potent and selective inhibitor of Bcl‐2, approved for treating chronic lymphocytic leukemia (CLL) and acute myeloid leukemia (AML) in patients with specific genetic profiles indicating Bcl‐2 dependence.^141^, ^158^ Venetoclax has demonstrated significant efficacy in inducing apoptosis in Bcl‐2‐dependent cancer cells, with a favorable safety profile compared with earlier, less specific Bcl‐2 inhibitors. However, cancer cells may develop resistance to BH3 mimetics by upregulating other antiapoptotic proteins like Mcl‐1. To overcome resistance, BH3 mimetics are often used in combination with other therapies, such as chemotherapy, targeted therapies, or other apoptosis‐inducing agents.^158^, ^159^

#### 4.1.2 Death receptor agonists

The extrinsic pathway of apoptosis is initiated by the binding of specific ligands to death receptors on the cell surface, which are part of the TNF receptor superfamily, including TRAIL receptors (DR4 and DR5) and Fas (CD95).^142^ Death receptor agonists are designed to activate these receptors and trigger apoptosis in cancer cells. TRAIL binds to death receptors DR4 and DR5, forming a DISC that recruits and activates caspase‐8, while Fas Ligand (FasL) binds to the Fas receptor (CD95), similarly leading to DISC formation and caspase‐8 activation. The activation of caspase‐8 initiates a proteolytic cascade, ultimately activating effector caspases such as caspase‐3, leading to apoptosis. Examples include recombinant TRAIL and agonistic antibodies targeting DR4 or DR5, which selectively induce apoptosis in cancer cells, and agonistic antibodies and recombinant FasL, which induce apoptosis in Fas‐expressing cancer cells.^143^, ^160^ Despite promising preclinical data, clinical trials with TRAIL and FasL agonists have encountered limited efficacy in some cancers due to intrinsic or acquired resistance mechanisms. Additionally, death receptor agonists can potentially cause toxicity in normal tissues expressing death receptors, leading to adverse effects. Advances in targeted delivery systems and combination therapies are being explored to enhance the specificity and efficacy of death receptor agonists.^144^, ^161^

**FIGURE 5:**
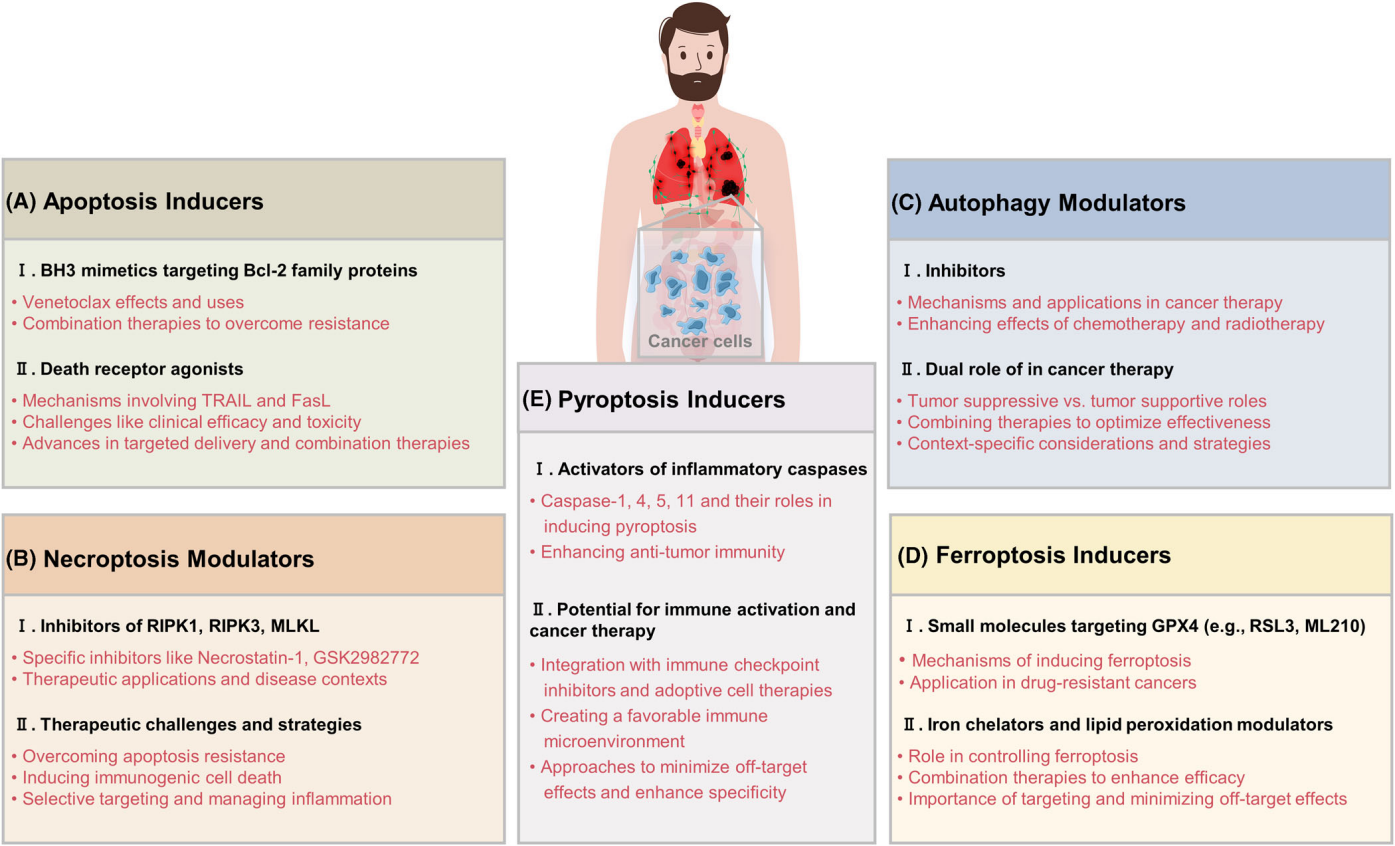
Therapeutic strategies targeting cell death pathways in cancer treatment. (A) Apoptosis pathways with two primary targets: the intrinsic pathway targeted by BH3 mimetics like Venetoclax which inhibit Bcl‐2 proteins, and the extrinsic pathway activated by death receptor agonists such as TRAIL and FasL that bind to death receptors DR4, DR5, and CD95, respectively, inducing apoptosis. (B) Necroptosis modulation, where the activation or inhibition of key proteins (RIPK1, RIPK3, MLKL) can trigger necroptosis, offering an alternative route to cellular demise distinct from apoptosis. (C) Autophagy and its dual role as both a tumor suppressor and a survival mechanism in cancer cells, illustrated using lysosomotropic agents such as chloroquine (CQ) and hydroxychloroquine (HCQ) which block the fusion of autophagosomes with lysosomes. (D) Ferroptosis inducers that manipulate iron metabolism and lipid peroxidation, highlighting small molecule inhibitors like RSL3 targeting GPX4. (E) Pyroptosis inducers and their role in stimulating an inflammatory response and enhancing antitumor immunity, featuring agents activating caspase‐1 and caspase‐4/5/11. Bcl‐2, B‐cell lymphoma‐2; DR4, death receptor 4; DR5, death receptor 5; CD95, death receptor 95; GPX4, glutathione peroxidase 4; MLKL, mixed lineage kinase domain‐like protein; RIPK1, receptor‐interacting serine/threonine‐protein kinase 1; RIPK3, receptor‐interacting serine/threonine‐protein kinase 3.

In conclusion, apoptosis inducers such as BH3 mimetics and death receptor agonists represent promising therapeutic strategies in cancer treatment (Figure 5A). By specifically targeting the dysregulated apoptotic pathways in cancer cells, these agents aim to selectively induce cell death and improve clinical outcomes. Ongoing research and clinical trials continue to refine these approaches, addressing challenges related to resistance, specificity, and safety.

### 4.2 Necroptosis modulators

Necroptosis is a form of programmed cell death distinct from apoptosis, characterized by cellular swelling, membrane rupture, and inflammation. It is regulated by key proteins including RIPK1, RIPK3, and MLKL.^162^ Unlike apoptosis, necroptosis is often associated with inflammatory responses, making it a double‐edged sword in disease contexts.

#### 4.2.1 Inhibitors of RIPK1, RIPK3, and MLKL

To modulate necroptosis for therapeutic benefit, researchers have developed inhibitors targeting the key proteins involved in the necroptosis pathway, specifically RIPK1, RIPK3, and MLKL.^145^ RIPK1 inhibitors block the kinase activity of RIPK1, preventing it from interacting with RIPK3 and initiating the necroptotic cascade. Examples include necrostatin‐1 (Nec‐1), one of the first RIPK1 inhibitors identified, which binds to the kinase domain of RIPK1, and GSK2982772, a more recent RIPK1 inhibitor currently under clinical investigation for inflammatory diseases.^146^, ^163^ RIPK3 inhibitors, such as GSK‐872, block the kinase activity of RIPK3, preventing it from phosphorylating and activating MLKL. Targeting RIPK3 may be beneficial in diseases where RIPK3‐mediated necroptosis plays a critical role, such as certain types of cancer and inflammatory conditions.^148^, ^149^ MLKL inhibitors, like Necrosulfonamide, prevent the oligomerization and membrane translocation of MLKL, thereby blocking the execution phase of necroptosis. Inhibition of MLKL might be useful in conditions where necroptosis contributes to pathology, such as inflammatory diseases.^150^

#### 4.2.2 Potential therapeutic applications and challenges

Overcoming apoptosis resistance in cancer therapy is a critical goal, and necroptosis presents a promising alternative by bypassing the resistance mechanisms that many cancers develop against apoptosis‐inducing therapies.^164^, ^165^ Inducing necroptosis can provide an alternative route to induce cancer cell death and may enhance overall efficacy when combined with traditional therapies such as chemotherapy and radiotherapy. Additionally, necroptosis is more immunogenic than apoptosis, leading to the release of DAMPs that can stimulate an antitumor immune response. This immunogenic cell death can potentially be combined with immune checkpoint inhibitors to amplify the immune system's ability to target and destroy cancer cells. Modulating necroptosis in the TME can also alter the inflammatory milieu, potentially making it less conducive to cancer growth, and targeting necroptosis in stromal cells can disrupt supportive niches for cancer cells. However, there are significant challenges in cancer therapy, including achieving selective targeting of necroptosis in cancer cells without affecting normal cells to avoid unintended tissue damage and inflammation. Additionally, cancer cells may develop resistance mechanisms to necroptosis modulators, and inhibiting necroptosis might activate alternative cell death pathways or survival mechanisms, reducing treatment efficacy (Figure 5B). The proinflammatory nature of necroptosis, while beneficial for stimulating an immune response, could also promote tumor growth and metastasis if not carefully managed. The context‐dependent effects of necroptosis in cancer, varying with tumor type, stage, and the specific TME, complicate the design of universal therapeutic strategies.^10^, ^166^, ^167^ Translating promising preclinical findings into clinical success requires rigorous clinical trials to establish the safety and efficacy of necroptosis modulators in cancer patients, and identifying patients most likely to benefit from necroptosis modulation necessitates the development and validation of biomarkers for necroptosis susceptibility.

### 4.3 Autophagy modulators

Autophagy is a cellular mechanism where damaged organelles and proteins are broken down and reused. It has a dual role in cancer, behaving as both a suppressor of tumor growth and a supporter of tumor survival depending on the circumstances. Modulating autophagy through inhibitors like chloroquine (CQ) and HCQ provides an intriguing approach to cancer therapy with both potential benefits and challenges.

#### 4.3.1 Autophagy inhibitors

CQ and HCQ are lysosomotropic agents that accumulate in lysosomes, increasing the pH and inhibiting lysosomal acidification, which disrupts the fusion of autophagosomes with lysosomes and blocks the final degradation step of autophagy. Both CQ and HCQ are United States Food and Drug Administration approved for malaria and certain autoimmune diseases, and their repurposing in cancer therapy is being actively explored.^151^ In cancer therapy, these autophagy inhibitors have potential applications in enhancing chemotherapy and radiotherapy, as cancer cells often upregulate autophagy in response to these treatments as a survival mechanism. Inhibiting autophagy can sensitize cancer cells to treatment‐induced death and potentially overcome resistance to targeted therapies and hormonal treatments in cancers such as breast cancer, prostate cancer, and melanoma.^168^, ^169^, ^170^ However, there are challenges and considerations, including the toxicity and side effects of long‐term use, such as retinal toxicity, which require careful monitoring and management in clinical settings. ^171^ Additionally, the role of autophagy in cancer is highly context dependent; in some cancers, autophagy can act as a tumor suppressor, and its inhibition might inadvertently promote tumor progression.^70^

#### 4.3.2 Dual role of autophagy in cancer therapy

Autophagy plays a dual role in cancer therapy as both a tumor suppressor and a tumor promoter. As a tumor suppressor, autophagy prevents tumorigenesis in the early stages by reducing oxidative stress and genomic instability through the removal of damaged organelles and proteins. The loss of autophagy‐related genes, such as Beclin 1, has been linked to increased tumorigenesis. Additionally, autophagy can induce type II programmed cell death, or autophagic cell death, under certain conditions, aiding in the elimination of cancer cells. Conversely, as a tumor promoter, established tumors exploit autophagy to survive under stress conditions like hypoxia, nutrient deprivation, and treatment‐induced stress, by providing metabolic substrates that support tumor growth and survival. This mechanism also contributes to therapeutic resistance, allowing cancer cells to adapt to and survive chemotherapy, radiotherapy, and targeted therapies.^172^, ^173^, ^174^ Therapeutic strategies to target autophagy must consider its context‐specific roles. Combination therapies, such as pairing HCQ with chemotherapy, can enhance treatment efficacy by preventing cancer cells from utilizing autophagy as a survival mechanism, which has shown promise in clinical trials for cancers like glioblastoma and pancreatic cancer.^175^, ^176^ Personalized medicine approaches, using biomarkers and genetic profiling, are essential for identifying patients who may benefit from autophagy inhibition.^177^ Optimizing timing and dosage through adaptive therapy can maximize benefits while minimizing adverse effects, with intermittent dosing schedules potentially reducing toxicity (Figure 5C). To address resistance mechanisms, combining autophagy inhibitors with inhibitors of other survival pathways, such as the PI3K/AKT/mTOR pathway, might enhance therapeutic efficacy and prevent resistance. Continuous monitoring of autophagy activity and adaptive treatment strategies are crucial for managing resistance and improving long‐term outcomes.^178^, ^179^

### 4.4 Ferroptosis inducers

Ferroptosis induction represents a promising strategy in cancer therapy, particularly for targeting drug‐resistant and aggressive cancers.^153^ Small molecules targeting GPX4, such as RSL3 and ML210, along with iron chelators and lipid peroxidation modulators, provide versatile tools to modulate ferroptosis. Careful selection of therapeutic combinations, targeting specific cancer types, and minimizing off‐target effects are essential for harnessing the full potential of ferroptosis in cancer treatment. Continued research and clinical development will be key to translating these strategies into effective therapies for patients.

#### 4.4.1 Small molecules targeting GPX4

Small molecules targeting GPX4 are emerging as effective inducers of ferroptosis in cancer therapy. GPX4 plays a critical role in protecting cells from lipid peroxidation, thereby inhibiting ferroptosis.^180^ RSL3, for instance, covalently binds to GPX4, inhibiting its activity and leading to the accumulation of LOOH, which ultimately triggers ferroptosis. RSL3 has shown efficacy in various cancer models, particularly those with high levels of ROS and lipid peroxidation, such as cancers with mutations in the Ras/Raf/MEK pathway that often exhibit elevated oxidative stress.^154^, ^181^ Similarly, ML210 is a potent and selective GPX4 inhibitor that disrupts the detoxification of lipid peroxides, leading to ferroptosis. Preclinical studies have demonstrated the promise of ML210, including its efficacy in models of drug‐resistant cancer cells, making it a valuable tool for studying ferroptosis and its therapeutic potential.^182^ Additionally, iron chelators and lipid peroxidation modulators play a crucial role in ferroptosis by influencing iron levels and lipid peroxidation. Iron, through the Fenton reaction, catalyzes the formation of lipid peroxides, and modulating these processes can impact ferroptosis and its application in cancer therapy.^183^, ^184^

#### 4.4.2 Iron chelators and lipid peroxidation modulators

Iron chelators and lipid peroxidation modulators play crucial roles in ferroptosis and its therapeutic application in cancer therapy. Iron chelators like deferoxamine and deferiprone bind free iron, reducing its availability and thereby inhibiting ferroptosis. In cancer therapy, iron chelators can prevent ferroptosis in normal cells, reducing off‐target effects and toxicity, and in some contexts, they can be combined with ferroptosis inducers to selectively target cancer cells with high iron content or to modulate the timing of ferroptosis induction.^155^ Lipid peroxidation modulators can either enhance or inhibit lipid peroxide formation. For example, liproxstatin‐1 is a ferroptosis inhibitor that prevents lipid peroxidation, while agents that promote lipid peroxidation can induce ferroptosis. Modulating lipid peroxidation provides a strategic approach to control ferroptosis; enhancing lipid peroxidation can synergize with GPX4 inhibitors to potentiate ferroptosis in cancer cells, whereas lipid peroxidation inhibitors can protect normal tissues, improving the safety profile of ferroptosis‐based therapies.^154^ Therapeutic strategies for ferroptosis induction include combination therapies that integrate ferroptosis inducers with other cancer treatments (e.g., chemotherapy, targeted therapy, immunotherapy) to enhance anticancer efficacy by overcoming resistance mechanisms and promoting synergistic cell death.^185^ For instance, combining GPX4 inhibitors with agents that increase ROS levels or inhibit other antioxidant pathways can potentiate ferroptosis and enhance cancer cell killing. Targeting susceptible cancers, particularly those with high oxidative stress or specific genetic mutations (e.g., Ras‐driven cancers), can maximize therapeutic benefits, with biomarker‐driven approaches helping to identify patients most likely to benefit from ferroptosis‐based therapies. Minimizing off‐target effects involves selecting appropriate iron chelators and lipid peroxidation modulators to protect normal cells, and developing targeted delivery systems, like nanoparticles or conjugates, to improve the specificity and efficacy of ferroptosis inducers.^186^, ^187^ Overcoming resistance to ferroptosis, which can arise through various mechanisms such as upregulation of antioxidant pathways or alterations in iron metabolism, is crucial for the success of ferroptosis‐based therapies. Combination strategies targeting multiple pathways involved in ferroptosis can help overcome resistance and improve treatment outcomes (Figure 5D).^164^, ^188^

### 4.5 Pyroptosis inducers

Pyroptosis induction represents a promising strategy for cancer therapy due to its ability to induce potent inflammatory cell death and enhance antitumor immunity.^156^ Activators of inflammatory caspases, such as caspase‐1 and caspase‐4/5/11, can induce pyroptosis, leading to tumor cell death and immune activation. The combination of pyroptosis inducers with immunotherapies, such as immune checkpoint inhibitors and adoptive cell therapies, holds significant potential for improving cancer treatment outcomes. Targeted delivery systems and prodrug approaches can further enhance the specificity and safety of pyroptosis‐based therapies, making them a viable option for overcoming resistance and enhancing the efficacy of existing cancer treatments.^157^

#### 4.5.1 Activators of inflammatory caspases

Activators of inflammatory caspases, particularly caspase‐1, caspase‐4, caspase‐5, and caspase‐11, hold significant therapeutic potential in cancer therapy by inducing pyroptosis and enhancing antitumor immunity. Activators of caspase‐1 can induce pyroptosis in cancer cells, leading to tumor cell death and the release of inflammatory cytokines that recruit and activate immune cells, thereby creating an immunogenic TME and potentially enhancing antitumor immunity. Activators of caspase‐4/5/11 can induce pyroptosis, particularly in cancer cells resistant to apoptosis or other forms of cell death, and enhance the immunogenicity of the tumor, promoting antitumor immune responses.^189^, ^190^, ^191^

#### 4.5.2 Potential for immune activation and cancer therapy

The potential for immune activation and cancer therapy through pyroptosis induction is significant, offering promising avenues for enhancing antitumor responses. Pyroptosis leads to the release of proinflammatory cytokines such as IL‐1β and IL‐18, which activate and recruit immune cells, including dendritic cells, macrophages, and T cells. This cytokine milieu enhances tumor antigen presentation and promotes a robust antitumor immune response. Additionally, the release of tumor antigens and danger signals during pyroptosis can enhance the activation and maturation of dendritic cells, improving antigen presentation to T cells and boosting adaptive immunity.^192^, ^193^ Combining pyroptosis inducers with immune checkpoint inhibitors (e.g., anti‐PD‐1, anti‐CTLA‐4) can increase tumor immunogenicity and overcome immune suppression in the TME, enhancing the efficacy of these therapies. Likewise, pyroptosis induction can be combined with adoptive cell therapies, such as CAR‐T cells or TCR‐engineered T cells, to create a more favorable immune microenvironment and enhance their effectiveness.^194^, ^195^ Pyroptosis provides an alternative cell death pathway that can be targeted in cancers resistant to apoptosis and can turn immunologically “cold” tumors into “hot” tumors, making them more responsive to immunotherapies. To minimize off‐target effects, targeted delivery systems like nanoparticle delivery or tumor‐specific promoters can specifically deliver pyroptosis inducers to tumor cells, reducing systemic inflammation. Prodrug approaches can also be used to activate pyroptosis inducers specifically within the TME, mitigating the risk of systemic toxicity (Figure 5E).^196^, ^197^

## 5 CLINICAL APPLICATIONS AND CHALLENGES

The exploration of cell death pathways, including apoptosis, necroptosis, and pyroptosis, has paved the way for innovative cancer therapies. This section focuses on the clinical applications and challenges associated with these therapeutic strategies, with a particular emphasis on ongoing and completed clinical trials.

### 5.1 Current clinical trials

#### 5.1.1 Overview of ongoing and completed trials

The clinical translation of therapies targeting cell death pathways is an active area of research, with numerous ongoing and completed clinical trials showcasing promising outcomes. Apoptosis inducers, such as BCL‐2 inhibitors, have shown significant results.^198^ Venetoclax (ABT‐199), particularly in hematological malignancies like CLL and AML, has demonstrated high response rates and improved progression‐free survival in CLL and encouraging responses in AML, especially in older patients.^199^, ^200^ Navitoclax (ABT‐263), targeting BCL‐2/BCL‐XL, has shown efficacy in combination with standard therapies in lymphoma and small cell lung cancer but has thrombocytopenia as a dose‐limiting toxicity due to BCL‐XL inhibition.^201^, ^202^ Necroptosis inducers, including RIPK1/RIPK3 inhibitors, are also under investigation.^146^ GSK2982772, a RIPK1 inhibitor, has been well tolerated in a Phase 1 trial with healthy volunteers, though further studies are needed to assess its efficacy in cancer.^203^ Nec‐1, another RIPK1 inhibitor, has shown tumor growth reduction in preclinical animal models and potential for combination therapy.^204^ Pyroptosis inducers, such as caspase‐1 activators, are being explored for their role in cancer therapy. VX‐765, a caspase‐1 inhibitor, demonstrated safety and tolerability in nononcology settings, and preclinical studies suggest its potential in cancer.^205^ Additionally, caspase‐4/5/11 activators like TAK‐242, a TLR4 inhibitor studied for sepsis, have shown effectiveness in reducing inflammatory responses and are being considered for repurposing in cancer therapy.^206^, ^207^ The key therapeutic agents targeting the cell death pathway in cancer clinical trials are shown in Table 2.

**TABLE 2:** The key therapeutic agents targeting cell death pathways in cancer clinical trials.

Representative drugs	Targets	Mechanisms	Cancer types	References
Venetoclax (ABT‐199)	BCL‐2	Inhibits BCL‐2 to induce apoptosis	Chronic lymphocytic leukemia (CLL), acute myeloid leukemia (AML)	199, 200
Navitoclax (ABT‐263)	BCL‐2/BCL‐XL	Inhibits BCL‐2 and BCL‐XL, inducing apoptosis	Lymphoma, small cell lung cancer (SCLC)	201
GSK2982772	RIPK1	RIPK1 inhibitor, potential to inhibit necroptosis	Under investigation in cancer (previously studied in inflammatory diseases)	203
Necrostatin‐1	RIPK1	Inhibits RIPK1, potentially blocking necroptosis	Under study in preclinical animal models for cancer	204
VX‐765	Caspase‐1	Inhibits caspase‐1, potentially affecting pyroptosis	Potential application in cancer therapy	205
TAK‐242	TLR4 (impacting Caspase‐4/5/11 indirectly)	Inhibits TLR4, reducing inflammatory responses, studied for potential repurposing in cancer	Considered for repurposing in cancer therapy	206

#### 5.1.2 Key findings and clinical outcomes

Key findings and clinical outcomes of therapies targeting cell death pathways underscore their efficacy and safety. Apoptosis‐inducing agents, particularly BCL‐2 inhibitors like venetoclax, have shown high response rates in hematological malignancies. Combining cell death pathway modulators with existing therapies, such as chemotherapy and immunotherapy, often enhances efficacy and overcomes resistance.^208^, ^209^ However, managing toxicity, such as thrombocytopenia with navitoclax, is crucial, necessitating dose adjustments and supportive care.^210^ Despite these advancements, challenges remain, including the development of resistance mechanisms by tumor cells, the need for selective targeting to minimize off‐target effects, and the identification of predictive biomarkers for patient selection and response monitoring. Future directions focus on identifying and validating novel targets within cell death pathways, combining cell death inducers with immunotherapies, targeted therapies, and conventional treatments to enhance therapeutic outcomes, and leveraging advances in genomics and biomarker discovery to refine patient selection and treatment customization, ultimately improving clinical outcomes.^211^, ^212^

### 5.2 Drug resistance mechanisms

Despite the promising therapeutic potential of targeting cell death pathways in cancer treatment, resistance to these therapies remains a significant hurdle.^213^ This section delves into the mechanisms by which cancer cells develop resistance to cell death‐targeting therapies and explores strategies to overcome this resistance. Resistance to cell death‐targeting therapies poses a significant challenge in the treatment of cancer.^213^ Understanding the mechanisms underlying this resistance is crucial for developing effective strategies to overcome it. Combination therapies, targeting antiapoptotic proteins, epigenetic modulation, autophagy inhibition, enhancing immune response, and precision medicine approaches represent promising strategies to counteract resistance and improve therapeutic outcomes.^214^, ^215^ Ongoing research and clinical trials will continue to refine these strategies and expand the arsenal of effective cancer treatments.

#### 5.2.1 Mechanisms of resistance to cell death‐targeting therapies

Genetic mutations and varied cellular mechanisms contribute significantly to resistance against therapies targeting cell death pathways. Mutations in BCL‐2 family proteins, such as BAX or BAK, can block the permeabilization of the mitochondrial outer membrane, an important step in apoptosis. Mutations in TP53, the gene responsible for p53, reduce the ability to trigger apoptosis in response to DNA damage and other cellular stress signals. Increased levels of antiapoptotic proteins such as BCL‐2 and MCL‐1 can bind to proapoptotic proteins, preventing apoptosis and making cells resistant to BCL‐2 inhibitors like venetoclax. Likewise, a decrease in proapoptotic proteins like BAX and BAK, or the absence of BH3‐only proteins like BIM, PUMA, and NOXA, disrupts the apoptotic process, leading to resistance.^216^, ^217^, ^218^ Cancer cells often upregulate autophagy in response to stress, including treatment with cell death inducers, providing a survival advantage through cytoprotective autophagy.^219^ Epigenetic modifications, such as DNA methylation and histone modification, can silence genes involved in apoptosis, necroptosis, and pyroptosis, further contributing to resistance.^220^, ^221^ Additionally, cancer cells can activate compensatory survival pathways like the PI3K/AKT/mTOR pathway or the NF‐κB pathway to counteract the effects of cell death‐targeting therapies.^222^

#### 5.2.2 Strategies to overcome resistance

Combination therapies offer promising strategies to enhance therapeutic efficacy and overcome resistance in cancer treatment.^223^, ^224^ Targeting multiple pathways by combining cell death inducers with agents that inhibit compensatory survival pathways, such as combining BCL‐2 inhibitors with PI3K inhibitors, can prevent the activation of alternative survival mechanisms. Synergistic drug combinations, like using BCL‐2 inhibitors with MCL‐1 inhibitors, target different components of the apoptotic pathway, effectively inducing apoptosis. Developing specific MCL‐1 inhibitors and dual inhibitors that target multiple antiapoptotic proteins simultaneously can further overcome resistance by preventing cancer cells from compensating through alternative pathways.^225^, ^226^, ^227^ Epigenetic modulation, using agents like DNA methylation inhibitors or histone deacetylase inhibitors, can restore the expression of proapoptotic genes and enhance cell death.^228^, ^229^ Inhibiting autophagy with agents such as CQ or HCQ, in combination with cell death inducers, can prevent the protective effect of autophagy and enhance cancer cell death.^230^, ^231^ Enhancing the immune response through immunotherapy, such as combining cell death‐targeting therapies with immune checkpoint inhibitors, can help eliminate resistant cancer cells via immune‐mediated mechanisms.^232^ Additionally, cancer vaccines or adoptive T cell therapies targeting tumor‐specific antigens released during cell death can bolster the immune response against resistant cells.^233^ Precision medicine approaches, including biomarker‐guided therapy and comprehensive genomic profiling, enable the identification of resistance biomarkers and genetic alterations, guiding the selection of appropriate combination therapies and personalized treatment regimens for individual patients.^234^

### 5.3 Combination therapies

The combination of cell death‐targeting therapies with other treatment methods like chemotherapy, radiotherapy, and immunotherapy has demonstrated potential in improving treatment effectiveness and overcoming resistance.^235^, ^236^ This section will explore the synergistic effects of combination therapies, their potential benefits, and the challenges involved. Table 3 lists the drug combinations and their synergistic effects in combination therapy in some cancers.

**TABLE 3:** The synergistic effects of combination therapies in cancer.

Drug 1	Drug 2	Mechanisms	Cancer types	References
Quercetin	Doxorubicin	Induction of apoptosis	Retinoblastoma	237
Berberine	Cinnamaldehyde	Induction of apoptosis and inhibition of proliferation, autophagy	Non‐small cell lung cancer	238
Berberine	Doxorubicin	Induction of apoptosis and decertation of cell viability	Non‐small cell lung cancer	238
Berberine	Osimertinib	Induction of apoptosis	Non‐small cell lung cancer	238
Berberine	TPD7	Induction of apoptosis	Leukemia	238
Berberine	Galangin	Induction of apoptosis	Esophageal cancer	238
Berberine	Cisplatin	Induction of apoptosis	Ovarian cancer	238
Berberine	Niraparib	Induction of apoptosis	Ovarian cancer	238
Berberine	Cisplatin	Induction of apoptosis and necroptosis	Ovarian cancer	238
Berberine	Doxorubicin	Induction of G2/M phase arrest and apoptosis	Melanoma	238
Berberine	Cisplatin and PDT	Induction of apoptosis	Melanoma	238
Neratinib	Ado‐Trastuzumab‐Emtansine	Inhibition of metastasis	Breast Cancer	239
HRS‐4642	Carfilzomib	Inhibition of KRAS G12D‐mutant cancer model	–	240
Temozolomide	Dasatinib/Rapamycin/Temozolomide	–	Neuroblastoma	241
Ramucirumab	pembrolizumab	Inhibition of metastasis	Head and neck squamous cell carcinoma	242
Celecoxib	Paclitaxel	Improved drug accumulation and triggered robust antitumor immunity	Triple‐negative breast cancer	243
Cetuximab	FOLFOXIRI/ FOLFOX	Inhibition of liver metastasis	Colorectal cancer	244

#### 5.3.1 Synergistic effects with chemotherapy, radiotherapy, and immunotherapy

Chemotherapy often induces cell death through DNA damage, disruption of replication, or inhibition of cellular division. When combined with cell death‐targeting agents, this dual assault can enhance apoptosis. For example, BCL‐2 inhibitors like Venetoclax, when combined with standard chemotherapy agents such as cytarabine, have demonstrated enhanced efficacy in AML.^245^ Similarly, PARP inhibitors, which target DNA repair mechanisms, can be combined with DNA‐damaging chemotherapies to enhance cancer cell death in BRCA‐mutated cancers.^246^, ^247^ Radiotherapy induces DNA damage and generates ROS, leading to cancer cell death. Cell death‐targeting agents can sensitize cancer cells to the effects of radiation. For instance, combining radiotherapy with apoptosis‐inducing agents like TRAIL receptor agonists can enhance the proapoptotic effects of radiation.^248^ Additionally, radiation can induce cytoprotective autophagy, and combining it with autophagy inhibitors can enhance the effectiveness of radiotherapy.^249^ Immunotherapies, such as immune checkpoint inhibitors (e.g., anti‐PD‐1/PD‐L1, anti‐CTLA‐4), enhance the immune system's ability to recognize and destroy cancer cells. Cell death‐targeting therapies can increase the release of tumor antigens, enhancing the immune response. For example, combining checkpoint inhibitors with apoptosis‐inducing agents can amplify the immune‐mediated killing of cancer cells, and cell death‐targeting therapies combined with adoptive T cell therapies can enhance the presentation of tumor antigens, improving T cell recognition and killing of cancer cells.^250^, ^251^, ^252^

#### 5.3.2 Potential benefits and challenges

Enhanced efficacy in cancer treatment can be achieved through combination therapies, which often produce synergistic effects, making the combined treatment more effective than the sum of individual therapies and overcoming resistance by targeting multiple pathways. This approach can lead to better treatment outcomes and potentially allow for lower doses of each agent, thereby reducing overall toxicity and side effects associated with high‐dose monotherapy.^253^ Additionally, combination therapies can target heterogeneous tumors more comprehensively by addressing different subpopulations of cancer cells within the tumor.^254^ Moreover, cell death‐targeting therapies can enhance the release of tumor antigens, improving the efficacy of immunotherapies and stimulating a more robust immune response.^51^ However, the complexity of treatment regimens poses significant challenges, including optimizing the timing, dosing, and sequencing of therapies to maximize synergy and minimize toxicity, as well as ensuring patient compliance, particularly for those with multiple comorbidities. Increased toxicity is another concern, as combining therapies can elevate the risk of adverse effects and off‐target interactions, necessitating close monitoring.^255^ Developing predictive biomarkers to identify which patients will benefit from specific combinations is crucial but challenging, requiring extensive research and validation. Tumors may also develop adaptive resistance mechanisms to combination therapies, necessitating ongoing research to counter evolving resistance patterns. Furthermore, the financial burden of combination therapies can be substantial, posing challenges to healthcare systems and patients, and ensuring equitable access to these treatments, particularly in resource‐limited settings, remains a significant issue.

## 6 FUTURE DIRECTIONS AND RESEARCH OPPORTUNITIES

The field of cancer therapy is rapidly evolving, driven by advances in our understanding of cell death mechanisms, personalized medicine, and drug delivery systems. This section outlines key future directions and research opportunities that hold promise for improving cancer treatment outcomes.

### 6.1 Identification of new cell death regulators

#### 6.1.1 High‐throughput screening and omics approaches

High‐throughput screening (HTS) and omics approaches are pivotal in advancing cancer therapy.^256^, ^257^ HTS technology enables the rapid testing of thousands to millions of compounds or genetic perturbations, facilitating the identification of new regulators of cell death. Applications of HTS include screening small molecules, CRISPR/Cas9 libraries, or RNA interference libraries to uncover novel drug targets and signaling pathways involved in cell death. For example, screening for compounds that selectively induce cell death in cancer cells but not in normal cells can lead to the discovery of new therapeutic agents with minimal side effects.^258^, ^259^, ^260^ Omics approaches, such as genomics, proteomics, and metabolomics, also play a crucial role. Genomics through whole‐genome sequencing and transcriptomics can identify mutations and gene expression changes associated with cell death regulation. Proteomics, using mass spectrometry, can reveal alterations in protein expression, posttranslational modifications, and protein‐protein interactions involved in cell death pathways. Metabolomics can identify changes in metabolic pathways that influence cell death, providing insights into the metabolic vulnerabilities of cancer cells. Integrating data from genomics, proteomics, and metabolomics can offer a comprehensive understanding of cell death mechanisms and identify potential therapeutic targets.^261^, ^262^, ^263^

#### 6.1.2 Novel biomarkers for therapy response

Predictive biomarkers play a crucial role in cancer therapy by guiding treatment selection and improving patient outcomes, with examples including BRCA mutations for PARP inhibitors and PD‐L1 expression for immune checkpoint inhibitors, which help identify patients likely to benefit from these therapies. Prognostic biomarkers provide information about the likely course of the disease, aiding in stratifying patients based on risk and tailoring treatment intensity. For instance, biomarkers indicating high levels of apoptosis or autophagy can inform prognosis and therapeutic strategies. Dynamic biomarkers, which change in response to treatment, offer real‐time feedback on therapeutic efficacy and guide adjustments in therapy. Examples include circulating tumor DNA (ctDNA) or specific protein markers in blood samples, which can be monitored to assess treatment response and detect early signs of resistance.^264^, ^265^, ^266^, ^267^

### 6.2 Personalized medicine

#### 6.2.1 Patient‐specific targeting of cell death pathways

Genetic profiling of tumors allows for the identification of mutations and alterations in cell death pathways unique to each patient, such as mutations in the TP53 gene, which plays a crucial role in apoptosis and can guide the use of therapies that exploit p53‐dependent cell death mechanisms.^268^, ^269^ Functional genomics approaches, including CRISPR screens, can identify essential genes and pathways in individual tumors, thereby guiding the selection of targeted therapies. For example, CRISPR screens can uncover synthetic lethal interactions, revealing vulnerabilities unique to specific genetic contexts and enabling personalized therapeutic strategies.^270^, ^271^, ^272^

#### 6.2.2 Tailoring treatments based on genetic and molecular profiles

Customized drug combinations, tailored to the specific genetic and molecular profile of a patient's tumor, can enhance efficacy and reduce toxicity. For instance, combining BCL‐2 inhibitors with other targeted therapies in patients with specific genetic alterations in apoptotic pathways can be particularly effective. Adaptive therapy involves adjusting treatment based on real‐time monitoring of tumor response and resistance patterns, thereby tailoring therapy to the evolving tumor landscape. An example of this approach is using ctDNA to monitor tumor evolution and adjust therapy accordingly, preventing the outgrowth of resistant clones.^273^, ^274^

### 6.3 Advances in drug delivery systems

#### 6.3.1 Nanotechnology and targeted delivery

Nanoparticle‐based delivery systems offer significant advantages by enhancing the delivery of therapeutic agents to tumor cells while minimizing off‐target effects. For example, liposomal formulations of chemotherapeutic agents, such as liposomal doxorubicin, improve drug accumulation in tumors and reduce systemic toxicity.^275^ Additionally, targeted delivery systems use ligands, antibodies, or peptides to direct therapeutic agents specifically to tumor cells. An example of this is antibody–drug conjugates, which combine a monoclonal antibody specific to a tumor antigen with a cytotoxic drug, thereby enhancing selective delivery to cancer cells.^276^, ^277^, ^278^

#### 6.3.2 Overcoming barriers to effective drug delivery

The TME, characterized by hypoxia, a dense ECM, and abnormal vasculature, poses significant challenges to drug delivery. Strategies to modify the TME, such as using agents that normalize tumor blood vessels or degrade the ECM, can enhance drug penetration. For example, combining VEGF inhibitors with chemotherapeutic agents can improve vascular normalization and drug delivery. Drug resistance, due to mechanisms like efflux pumps and drug metabolism, also reduces drug efficacy. Strategies to overcome resistance include co‐delivering efflux pump inhibitors or using prodrugs that are specifically activated within the tumor environment. An example is prodrugs activated by enzymes overexpressed in tumors, such as MMPs, ensuring selective activation within the tumor.^279^, ^280^, ^281^

All in all, the future of cancer therapy lies in the integration of HTS, omics approaches, personalized medicine, and advanced drug delivery systems. Identifying new cell death regulators and developing novel biomarkers will enable more precise targeting of therapies. Personalized medicine approaches, tailored to the genetic and molecular profiles of individual patients, promise to enhance treatment efficacy and minimize toxicity. Advances in drug delivery systems, including nanotechnology and targeted delivery, will overcome current barriers and improve therapeutic outcomes. Continued research and innovation in these areas are essential to realize the full potential of these strategies in cancer treatment.

Despite the remarkable advancements in cancer therapy technologies, such as HTS, omics approaches, personalized medicine, and advanced drug delivery systems, significant challenges and limitations persist in their practical applications. A primary concern is the high cost and complexity associated with these technologies, which can restrict accessibility and scalability in clinical settings, particularly in low‐resource environments. Additionally, the variability in individual patient responses presents a substantial challenge in effectively tailoring and predicting treatment outcomes through personalized medicine. Moreover, technological limitations in accurately modeling and predicting complex biological systems may result in unforeseen consequences and reduced efficacy of targeted therapies. Finally, regulatory and ethical issues surrounding the use of genetically tailored treatments, along with the potential for unintended off‐target effects, pose critical concerns that must be addressed to ensure the safe and equitable implementation of these emerging technologies in cancer treatment. These challenges highlight the necessity for ongoing research, technological improvements, and the development of frameworks to effectively manage these issues.

## 7 CONCLUSION

Over the course of this review, we have undertaken a comprehensive elucidation of the principal cell death pathways‐apoptosis, necroptosis, autophagy, ferroptosis, and pyroptosis and their intricate interplay in cancer development and therapy. Each pathway exhibits unique characteristics and molecular mechanisms that not only determine cell fate but also significantly influence the TME and the efficacy of cancer therapies.

Apoptosis remains a focal point due to its well‐established roles and therapeutic targets, involving critical regulators such as the Bcl‐2 protein family, caspases, and death receptors like Fas and TRAIL.^25^ The shift from viewing necrosis solely as an uncontrollable form of cell death to understanding necroptosis as a regulated, programmable phenomenon has opened new avenues for therapy, particularly against tumors resistant to traditional apoptotic triggers.^50^ Similarly, autophagy's dual role as both a survival mechanism and a modality of cell death underscores its complex relationship with cancer, necessitating a nuanced therapeutic approach.^282^ Emerging research into ferroptosis and pyroptosis has revealed these forms of cell death as crucial players in the cancer landscape, driven respectively by iron‐dependent lipid peroxidation and inflammation‐associated mechanisms.^94^ The interactions of these pathways with oxidative stress and inflammatory responses provide fertile ground for novel therapeutic strategies that might exploit these vulnerabilities in cancer cells.

The therapeutic implications of targeting cell death pathways are vast. Encouragingly, clinical trials exploring agents like BH3 mimetics and death receptor agonists have shown promise.^283^, ^284^ However, challenges persist, especially regarding resistance mechanisms and the selective induction of specific types of cell death in cancer cells without harming normal tissues. The therapeutic modulation of autophagy, which plays ambivalent roles in cancer, represents a particularly complex but potentially rewarding strategy. Additionally, the inducement of necroptosis and ferroptosis in situations where apoptosis is circumvented offers a strategic bypass to resistance, a recurrent issue in many therapeutic regimes.^285^ Future studies are essential to further delineate the cross‐talk between different cell death pathways and their interactions with the TME. HTS and omics technologies promise to unearth new regulators of cell death and provide insights that could lead to more robust, targeted therapies. Moreover, the integration of these findings with data from genomics and proteomics will enhance the development of personalized medicine approaches, aiming to tailor treatments to the specific molecular profiles of patients’ tumors.^286^, ^287^

In conclusion, the complexity of cell death processes and their modulation by various internal and external factors in cancer offers a rich panorama of research and therapeutic opportunities. Innovative therapeutics that can specifically initiate or block particular pathways, according to the oncogenic context, stand to revolutionize the management of cancer. Continuing to unravel the molecular tapestry of cell death will undoubtedly refine our strategies to fight cancer, transforming the prospects of clinical outcomes. As we move forward, the fusion of traditional research with cutting‐edge technologies will catalyze the next wave of advancements in cancer therapy, potentially turning the tide against this formidable disease.

## AUTHOR CONTRIBUTIONS

Shaohui Wang, Yeke Wu, and Yi Zhang conceptualized the manuscript. Shaohui Wang, Jing Guo, and Sa Gua wrote the first draft of the manuscript. Qinyun Du and Cen Wu contributed to the revision of the manuscript. All authors agreed and approved the final manuscript.

## CONFLICT OF INTEREST STATEMENT

The authors declare no conflict of interest.

## ETHICS STATEMENT

Not applicable.

## Data Availability

Not applicable.
